# A Genome-Wide Association Study Identifies Multiple Regions Associated with Head Size in Catfish

**DOI:** 10.1534/g3.116.032201

**Published:** 2016-08-24

**Authors:** Xin Geng, Shikai Liu, Jun Yao, Lisui Bao, Jiaren Zhang, Chao Li, Ruijia Wang, Jin Sha, Peng Zeng, Degui Zhi, Zhanjiang Liu

**Affiliations:** * Fish Molecular Genetics and Biotechnology Laboratory, Aquatic Genomics Unit, School of Fisheries, Aquaculture and Aquatic Sciences, Auburn University, Alabama 36849; † Department of Mathematics and Statistics, Auburn University, Alabama 36849; § Department of Biostatistics, University of Alabama at Birmingham, Alabama 35294; ‡ School of Biomedical Informatics, the University of Texas Health Science Center at Houston, Texas 70030; ** School of Public Health, the University of Texas Health Science Center at Houston, Texas 77030

**Keywords:** head size, GWAS, QTL, fish, hybrid, GenPred, shared data resources, genomic selection

## Abstract

Skull morphology is fundamental to evolution and the biological adaptation of species to their environments. With aquaculture fish species, head size is also important for economic reasons because it has a direct impact on fillet yield. However, little is known about the underlying genetic basis of head size. Catfish is the primary aquaculture species in the United States. In this study, we performed a genome-wide association study using the catfish 250K SNP array with backcross hybrid catfish to map the QTL for head size (head length, head width, and head depth). One significantly associated region on linkage group (LG) 7 was identified for head length. In addition, LGs 7, 9, and 16 contain suggestively associated regions for head length. For head width, significantly associated regions were found on LG9, and additional suggestively associated regions were identified on LGs 5 and 7. No region was found associated with head depth. Head size genetic loci were mapped in catfish to genomic regions with candidate genes involved in bone development. Comparative analysis indicated that homologs of several candidate genes are also involved in skull morphology in various other species ranging from amphibian to mammalian species, suggesting possible evolutionary conservation of those genes in the control of skull morphologies.

Skull morphology and body conformation are fundamental to evolution and the biological adaptation of species to their environments. Species evolve to have different head shapes and sizes in response to their environments, and in relation to their behavior and mode of survival. As such, skull morphology and body conformation have been extensively studied in various species. Wunnenberg-Stapleton *et al.* (1999) first reported the involvement of small GTPases (Ras homolog gene family member A and Ras homolog gene family member S) in the control of head formation in *Xenopus*. Later, a number of studies were conducted in canines. As a companion species, dogs have been artificially bred and selected to have hundreds of breeds, with various overall sizes and various head shapes and sizes (Schoenebeck and Ostrander 2013). The finding that selection of a single gene, *insulin-like growth factor I*, is largely responsible for the huge variations in shapes and sizes in dogs astonished many scientists (Sutter *et al.* 2007). Since then, great efforts have been devoted to the analysis of head shapes and sizes in dogs in order to understand the genomic basis underlying the large differences (Schoenebeck and Ostrander 2013). Genome-wide association studies (GWAS) have allowed the mapping of quantitative trait loci (QTL) controlling head shapes in eight chromosomes in dogs. As the canine reference genome is available, further analysis of the QTL regions has allowed the identification of candidate gene *bone morphogenetic protein 3* (*BMP3*) for skull shapes (Schoenebeck *et al.* 2012). Recently, seven QTL were identified for skull size and 30 QTL were identified for skull shape in mice (Maga *et al.* 2015).

With aquaculture fish species, analysis of head sizes is important not only for understanding evolution and biological adaptation, but also for economic reasons. Head shapes and sizes influence fillet yield directly. Smaller head and uniform body conformation provide a greater proportion of fillet, thus selection for smaller head and uniform body conformation is of great aquaculture value (Rutten *et al.* 2005). For example, compared with blue catfish, channel catfish has a relatively larger head and lower fillet yield ratio (Argue *et al.* 2003).

QTL linkage mapping analysis of body shape has been conducted in fish species including common carp (Laghari *et al.* 2014) and sea bass (Massault *et al.* 2010). However, limited by the number of markers, these findings are far from the requirements of marker-assisted selection (MAS), and little is known about genetic mechanisms for head shapes and sizes with aquaculture species. Channel catfish (*Ictalurus punctatus*) is the major aquaculture species in the United States. In recent years, hybrid catfish, produced by mating female channel catfish with male blue catfish (*I. furcatus*), has become the breed of choice because the F1 hybrid exhibits a number of superior traits due to heterosis including: faster growth, enhanced disease resistance, and greater fillet yield (Argue *et al.* 2003; Dunham *et al.* 2008). Channel catfish in general has a relatively larger head than blue catfish. Therefore, the channel catfish × blue catfish hybrid system offers a great model to study head shapes and sizes. Understanding of genomic regions for head shapes and sizes in catfish would allow us to discover controlling mechanisms. In addition, the linked markers will allow MAS or marker-guided introgression. Although traditional selective breeding has been used to enhance processing yield in catfish, the progress has been limited due to low selection intensity, low accuracy, and low heritability (Argue *et al.* 2003).

GWAS has been regarded as a powerful strategy for the identification of markers associated with traits of interest with high resolution, and it has been conducted in aquaculture species recently (Geng *et al.* 2015; Ayllon *et al.* 2015; Tsai *et al.* 2015, 2016; Sodeland *et al.* 2013; Correa *et al.* 2015, 2016; Jin *et al.* 2016; Gutierrez *et al.* 2015). Recent development of a number of genomic resources has made such work feasible in catfish, including the channel catfish reference genome sequence (Liu *et al.* 2016), a large number of SNPs (Sun *et al.* 2014), and the 250K SNP array (Liu *et al.* 2014). In this study, we explored the genetic architecture for catfish head size using GWAS, and here we report the identified QTL and the genes within the highly associated genomic regions.

## Materials and Methods

### Ethics statement

All experiments involving the handling and treatment of fish were approved by the Institutional Animal Care and Use Committee (IACUC) at Auburn University. Blood was collected after euthanasia. All animal procedures were carried out according to the Guide for the Care and Use of Laboratory Animals and the Animal Welfare Act in the United States.

### Experimental fish and sample collection

The study population was the Auburn University 1-yr-old catfish generated from backcross of male F1 hybrid catfish (female channel catfish × male blue catfish) with female channel catfish. The female channel catfish were collected from the Marion strain (Dunham and Smitherman 1984), including the female parents of the F1 hybrid catfish. The population consisted of nine families (Supplemental Material, Figure S1 and Table S1). The offspring were mixed for communal culture. In order to assign the fish to each family, cluster analysis was conducted according to the IBS kinship matrix based on their genotypes (Geng *et al.* 2015). In total, 556 fish (average body weight 50.8 g ranging from 13 to 180 g) were randomly obtained from Auburn University Fish Genetics Facility and blood samples were collected. The head size, including head length, head width, and head depth, was measured at one time-point as the trait of interest (Figure S2). Head length is the horizontal distance between the maxillary symphysis and the posterior bony edge of the operculum. Head width is the distance between the two sides of the posterior bony edges of the operculum. Head depth is the vertical distance from top to bottom of the skull across the posterior bony edge of the operculum.

### DNA isolation, genotyping, and quality control

DNA was isolated from blood samples using standard protocols. After incubated at 55° for ∼10 hr, the blood cells were broken by cell lysis solution. Protease K and protein precipitation solution were used to remove the proteins. Next, DNA was precipitated by isopropanol and collected by brief centrifugation, washed twice with 70% ethanol, air-dried, and resuspended in TE buffer (pH 8.0). After being quantified using spectroscopy by Nanodrop (Thermo Scientific) and checked by 1% agarose gel electrophoresis stained with ethidium bromide for integrity, DNA was diluted to 50 ng/μl.

A catfish 250K SNP array has been developed using Affymetrix Axiom genotyping technology with markers distributed across the catfish genome at an average interval of 3.6 kb (Liu *et al.* 2014). Genotyping using the catfish 250K SNP array was performed at GeneSeek (Lincoln, Nebraska). No sample was excluded due to low quality or low call rate (<95%). 218,784 SNPs were kept after filtering out SNPs with genotyping error based on Mendelian laws, a minor allele frequency (MAF) <5%, or a call rate <95%.

### Statistical analysis

To determine which individual SNPs were associated with head size, a single SNP test was performed on all markers. Statistical analysis was carried out using the SVS software package (SNP & Variation Suite, Version 8.0) and PLINK (Version 1.07) (Purcell *et al.* 2007). The threshold P-value for genome-wide significance was calculated using Bonferroni correction based on the estimated number of independent markers and linkage disequilibrium (LD) blocks. LD pruning was conducted with a window size of 50 SNPs, a step of 5 SNPs, and *r*^2^ threshold of 0.2. Assuming each LD block represents one independent set of markers, the number of independent SNPs and LD blocks was 14,391. The significance level for genome-wide significance was set as 0.05 / 14,391 = 3.47e^−6^ [−log_10_(P-value) = 5.46] based on Bonferroni correction. The threshold of −log_10_(P-value) for suggestive association was arbitrarily set as 5. To visualize the sample structure, principal component analysis with the independent SNP markers was conducted and the plots representing the sample structure were constructed with the first three principal components (Figure S1).

A two-step GWAS procedure was performed. First, to eliminate the effect of body weight and between-family phenotypic stratification, the phenotypic data in the backcross population were adjusted with cubic root of body weight by simple linear regression within each family (Froese 2006). Second, the residuals were used as adjusted phenotypes to carry out GWAS. Two methods were utilized to compare their performance in this step. The first method was Efficient Mixed-Model Association eXpedited (EMMAX) analyses (Kang *et al.* 2010). It was conducted in SVS with the first four principal component scores of each sample as covariates. The model is listed as follows:

Y=Xb+Zu+e

where ***Y*** is the vector of phenotype; ***X*** is a matrix of fixed effects and ***b*** is a coefficient vector; ***Z*** is a matrix relating the instances of the random effect to the phenotypes, ***u*** is a vector representing the coefficients, Var(***u***) = σ^2^_g_***G*** where σ^2^_g_ is the additive genetic variance and ***G*** is the genomic kinship matrix; and ***e*** is the vector of random residuals. This method models phenotypes using a mixture of fixed and random effects. Fixed effects include the SNPs and optional covariates, while random effects include heritable (***Zu***) and nonheritable random (***e***) variation (Price *et al.* 2010). Heritability was estimated as *h*^2^ = σ^2^_g_ / σ^2^_p_ (σ^2^_p_ is the phenotype variance). The ratio of phenotypic variance explained by one QTL is *R*^2^ = σ^2^_QTL_ / σ^2^_p_ (σ^2^_QTL_ is effect variance of this QTL). The second method was the family-based association test for quantitative traits (QFAM) conducted in PLINK (Fulker *et al.* 1999; Abecasis *et al.* 2000; Purcell *et al.* 2007). It breaks down the genotypes into between-family (b) and within-family (w) components, and the latter is free of population structure (Abecasis *et al.* 2000). Label the marker genotype score for the jth offspring in the ith family as g_*ij*_. The model is

$${\hat{y}}_{\text{ij}}\mathrm{=\mu}+\beta_{b}b_{i}+\beta_{w}w_{\mathrm{ij}}$$

where y_ij_ is the phenotype of individual *j* in family *i*; β_b_ is the coefficient of between-family effect and β_w_ is the coefficient of within-family effect; b_i_ = (∑g_ij_)/n_i_ if parental genotypes are unknown, and b_i_ = (g_iF_ + g_iM_)/2 if parental genotypes are available (g_iF_: genotype of father, g_iM_: genotype of mother); w_ij_ = g_ij_ − b_i_.

Manhattan plots were produced using the SVS software. The genetic marker map was constructed according to channel catfish genome sequence (version Coco1.0, Liu *et al.*, unpublished results), since genome architectures of channel catfish and blue catfish are extremely similar with the same sets of parallel chromosomes according to our former studies and unpublished data (Liu *et al.* 2003; Kucuktas *et al.* 2009; Ninwichian *et al.* 2012). The data sets including the phenotypes, genotypes, and marker information are available at https://figshare.com/s/7ad1c3f2a3d3cca9fcbe, which are bed files for analyses in PLINK and SVS.

### Sequence analysis

The ±1 Mb regions surrounding the most significant SNP out of each QTL were examined for candidate genes according to their locations and functions. The genes within the genomic regions were predicted using FGENESH (Solovyev *et al.* 2006) and annotated by BLAST analysis against the nonredundant protein database (Pruitt *et al.* 2007; Altschul *et al.* 1990).

### Data availability

The authors state that all data necessary for confirming the conclusions presented in the article are represented fully within the article.

## Results

### Phenotypes

A summary of the original observations and adjusted phenotypes for head length, head width, and head depth is shown in Table 1. Phenotypes were not normally distributed because the phenotypes segregated in the backcross generation. To cover individuals with a wide range of body size, samples were utilized with body weights varying from 13 to 180 g. Body weight could explain over 70% variance for all the three traits based on the coefficient of determination of linear regression.

**Table 1:** Summary of original observation and adjusted phenotype for three traits

	Original Observation				Adjusted Phenotype			
	Mean	SD	Min	Max	Mean	SD	Min	Max
Body weight (g)	50.8	23.9	13	180	—	—	—	—
Head length (cm)	3.36	0.49	1.97	5.16	0	0.23	−0.77	0.51
Head width (cm)	2.47	0.39	1.44	4.10	0	0.16	−0.75	0.47
Head depth (cm)	2.28	0.41	1.23	3.75	0	0.22	−0.65	0.83

*N* = 556. Min, minimum; Max, maximum.

### Determination of optimal model for analysis: EMMAX *vs.* QFAM

**Figure 1:**
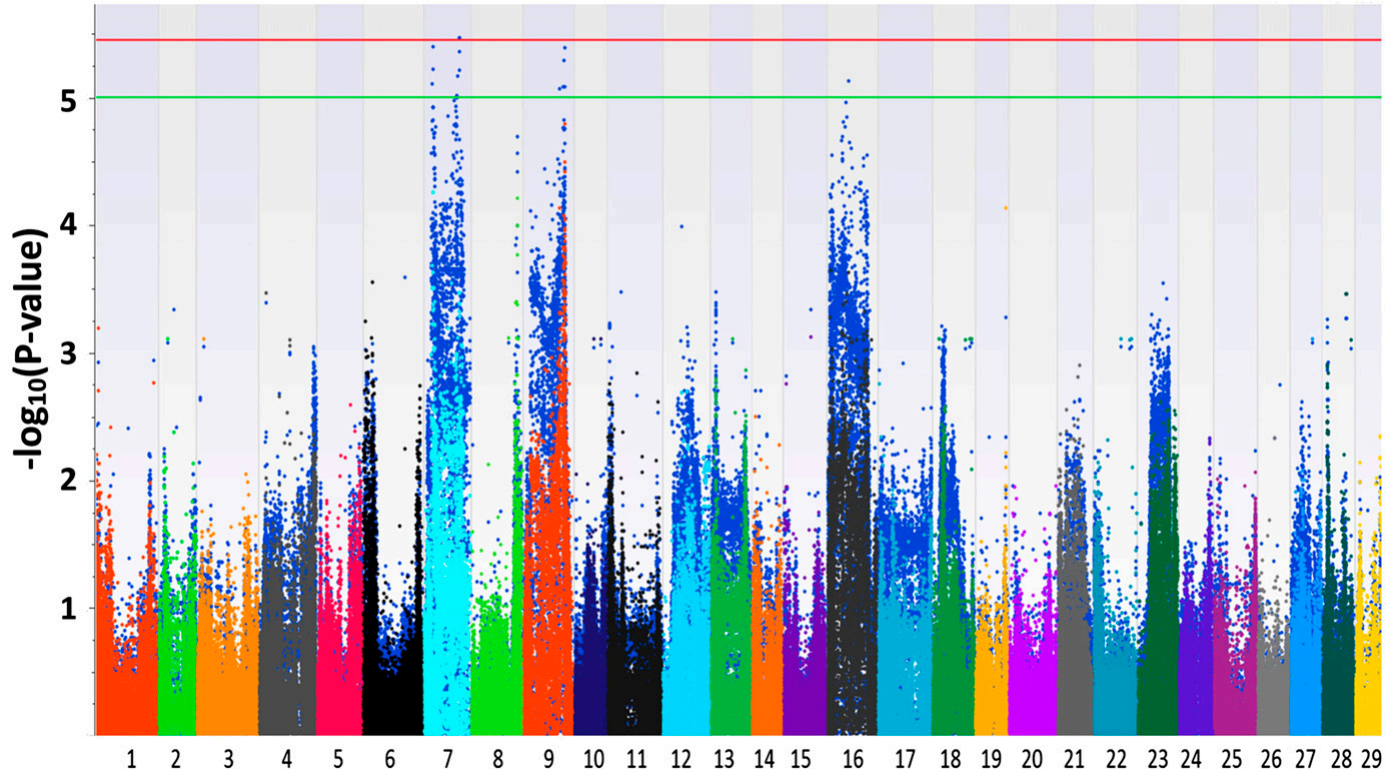
Manhattan plots for head length. The plots in different colors in the front layer were generated from EMMAX (Efficient Mixed-Model Association eXpedited) and the plots in blue in the back layer were generated from QFAM (family-based association test for quantitative traits).

**Figure 2:**
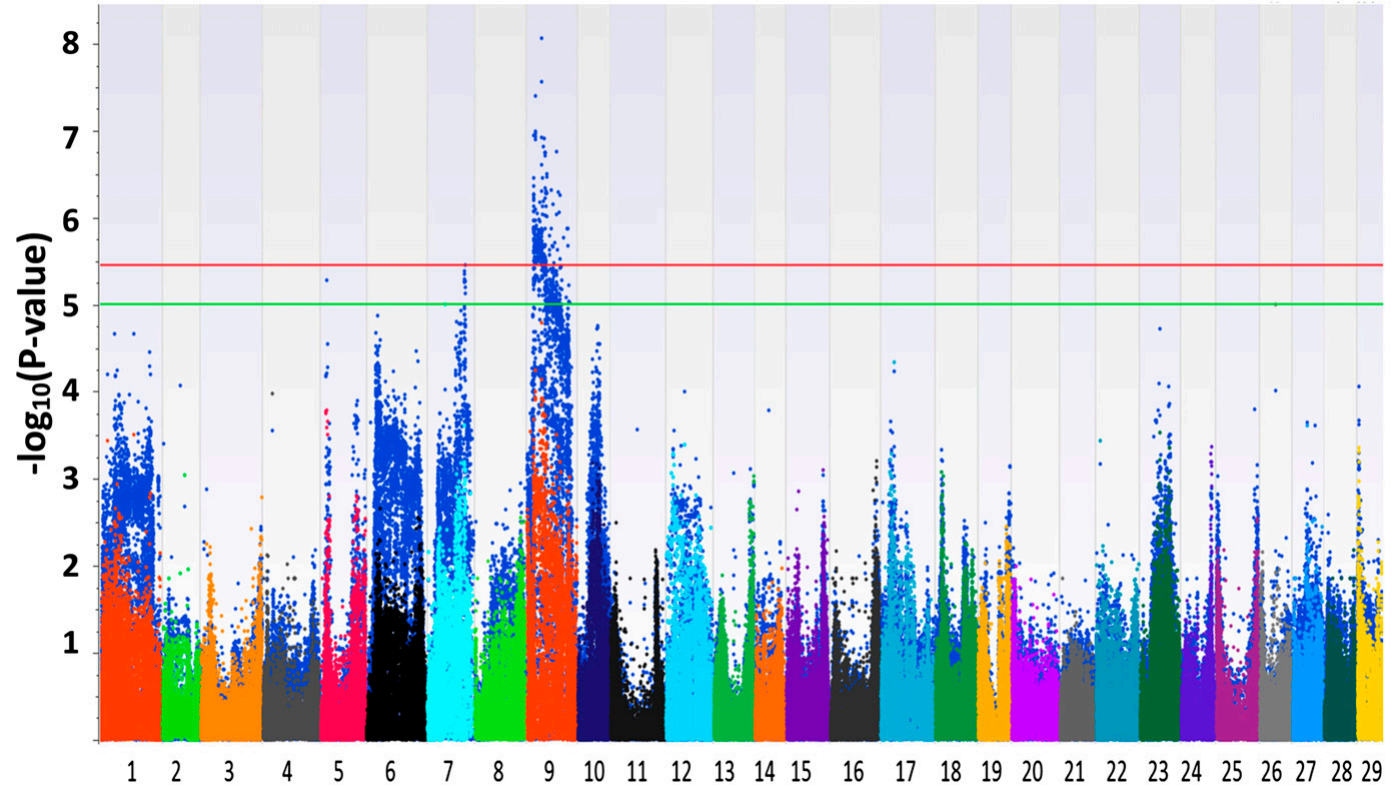
Manhattan plots for head width. The plots in different colors in the front layer were generated from EMMAX (Efficient Mixed-Model Association eXpedited) and the plots in blue in the back layer were generated from QFAM (family-based association test for quantitative traits).

The Manhattan plots generated from EMMAX and QFAM are shown in Figure 1 and Figure 2. Both of the methods could correct family structure efficiently. Generally, the association results generated by EMMAX and QFAM were positively correlated, but QFAM provided more statistical power than EMMAX with our family-based samples. In the following sections, we will describe the characters of identified regions according to the results generated from QFAM.

### Genomic regions associated with head size

For head length, one significantly associated SNP was identified around position 22 Mb of LG7 (Figure 1 and Table 2). The −log_10_(P-value) of the most significant SNP reached 5.46. Three LGs (LG7, LG9, and LG16) were found to contain QTL suggestively associated with head length (Figure 1, Figure S3, and Table 2). For head width, LG9 harbored significant SNPs (Figure 2, Figure S4, and Table 3). Two more regions on LG5 and LG7 were suggestively associated with head width (Table 3). For head depth, no SNP with −log_10_(P-value) > 5 was identified (Figure S5). Several associated regions could extend a long distance. It may be caused by two or more candidate genes with a long interval that were located in the associated regions, just like the case shown in Figure S3, Table 2, and Table 3. Another reason may be that the recombination rate to break the linkage of nearby loci in the backcross progenies is low.

**Table 2:** Information of regions associated with head length

LG	SNP ID	SNP Position	β	−log_10_(P)	% Variance	Gene Position (kb)	Gene Name	Reference
7	85385268	21742476	0.08	5.46	2	21,084–21,109	*cad22l**^a^*	Wunnenberg-Stapleton *et al.* 1999; Marie 2002
						21,633–21,638	*cyp24a1**^a^*	St-Arnaud and Naja 2011
						21,936–21,939	*rgs9bp**^a^*	Homme *et al.* 2003; Thirunavukkarasu *et al.* 2002
						21,997–22,012	*syt homolog*	Zhao *et al.* 2008
						22,240–22,250	*smpd2**^a^*^,^*^b^*	Aubin *et al.* 2005; Tomiuk *et al.* 1998
	85285134	5099134	0.10	5.40		4477–4715	*pcdh9**^a^*	Wunnenberg-Stapleton *et al.* 1999; Unterseher *et al.* 2004
						5920–5934	*rgs8**^a^*^,^*^b^*	Homme *et al.* 2003; Thirunavukkarasu *et al.* 2002
						5946–5948	*rgs16**^a^*^,^*^b^*	Homme *et al.* 2003; Thirunavukkarasu *et al.* 2002
						5975–5995	*rgs5**^a^*^,^*^b^*	Homme *et al.* 2003; Thirunavukkarasu *et al.* 2002
						6015–6019	*rgs4**^a^*^,^*^b^*	Homme *et al.* 2003; Thirunavukkarasu *et al.* 2002
9	85413092	25028079	0.11	5.38	3	24,916–24,918	*fgfp1**^a^*^,^*^b^*	Szebenyi and Fallon 1998; Deng *et al.* 1996
						24,925–24,927	*fgfp2a**^a^*^,^*^b^*	Szebenyi and Fallon 1998; Deng *et al.* 1996
						25,001–25,019	*tapt1**^a^*	McPherron *et al.* 1999; Howell *et al.* 2007
						25,226–25,228	*fstl5**^a^*	Sidis *et al.* 2002
						25,600–25,627	*rpgf2**^a^*	Quilliam *et al.* 2002
	86013630	21887313	−0.11	5.07		21,984–22,010	*smpd3**^a^*^,^*^b^*	Aubin *et al.* 2005; Tomiuk *et al.* 1998
						22,112–22,134	*sept7**^a^*	Cao *et al.* 2007
						22,301–22,304	*rab33a**^a^*^,^*^b^*	Itzstein *et al.* 2011
16	86048455	12450095	−0.10	5.13	2	11,590–11,625	*asap2**^a^*	Myers and Casanova 2008
						11,630–11,634	*itbp1**^a^*	Ross *et al.* 1993
						12,603–12,626	*kif3b**^a^*	Schoenebeck *et al.* 2012; Maga *et al.* 2015
						12,651–12,656	*rab10**^a^*^,^*^b^*	Itzstein *et al.* 2011

LG, linkage group; SNP, single nucleotide polymorphism; ID, identifier.

a Means the candidate genes are small GTPase or related to small GTPases in function.

b Means the paralogs of the candidate genes were identified.

**Table 3:** Information of regions associated with head width

LG	SNP ID	SNP Position	β	−log_10_(P)	% Variance	Gene Position (kb)	Gene Name	Reference
9	85362293	8919023	0.09	8.06	3	8993–9042	*snx25**^a^*	Parks *et al.* 2001
	85220262	4755583	−0.09	7.40		4034–4038	*pcdh20**^a^*^,^*^b^*	Unterseher *et al.* 2004; Wunnenberg-Stapleton *et al.* 1999
						4058–4061	*rab9b**^a^*^,^*^b^*	Itzstein *et al.* 2011
						4498–4522	*ablm3*	Dos Remedios *et al.* 2003
7	85994489	22981883	0.06	5.45	2	22,522–22,536	*nudt3*	Shears *et al.* 2011
						23,035–23,041	*gnb1b**^a^*	Gong *et al.* 2010; Quarles and Siddhanti 1996
						23,161–23,168	*gnai2b**^a^*	Gong *et al.* 2010; Quarles and Siddhanti 1996
						23,547–23,552	*gnat1**^a^*	Gong *et al.* 2010; Quarles and Siddhanti 1996
5	85206630	3471449	−0.10	5.27	3	3913–3940	*itb3b**^a^*	Ross *et al.* 1993
						3942–3956	*synpo2l**^a^*	Asanuma *et al.* 2006
						4203–4208	*galr2*	McDonald *et al.* 2007

LG, linkage group; SNP, single nucleotide polymorphism; ID, identifier.

a Means the candidate genes are small GTPases or related to small GTPases in function.

b Means the paralogs of the candidate genes were identified in the regions associated with head length.

### Candidate genes within the QTL regions for head size

The ±1 Mb regions around the most significant SNP of each QTL were examined for genes within the genomic regions. Candidate genes for head length on the associated regions were identified with known function related to bone development (Table 2). Interestingly, paralogs of the candidate genes were identified within the associated regions in catfish, including genes coding for small GTPase, sphingomyelin phosphodiesterase, fibroblast growth factor-binding protein, and regulator of G-protein signaling (Table 2).

Some candidate genes for head width on LGs 5, 7, and 9 are also involved in bone development (Table 3). Some paralogs of candidate genes for head length were identified in the regions associated with head width as well, including *protocadherins 20* and *Ras-related protein Rab 9b*. Of course, the other genes within associated QTL might have unknown related functions, and they cannot be excluded as potential functional units affecting head size.

### Ratio of phenotypic variance explained by associated SNPs

The ratio of phenotypic variance explained by associated SNPs of head size in our population was estimated by EMMAX. Because of high correlation among SNPs on the same LG, only the most significant SNP on each associated LG was chosen for analyzing the fraction of phenotypic variance explained by the QTL. Thus, the ratio of phenotypic variance explained may be underestimated. Moreover, the calculated fraction may change with different population or sample sizes. The fraction of variance of head length that could be explained by the most significant SNP on LG7 is 0.02. In addition, the other suggestively associated regions could explain 0.05 in total (Table 2). The proportion of explained head width variance was 0.08 in total from significantly associated QTL and suggestively associated QTL (Table 3).

### Conditioned analysis results

Conditioned analyses were conducted to examine the correlation of the SNPs associated with head size. The association test was conducted with the most significant SNPs on each LG as a covariate (one SNP at a time). Because of the lack of recombination among SNPs on the same LG in the backcross population, the −log_10_(P-value) of SNPs on the same LG with the SNP included as covariate dropped drastically after conditioning, implying that the SNPs on the same LG were highly correlated. For example, after the most significant SNP for head length on LG9 (ID 85413092) was included in the association test, the −log_10_(P-value) of SNPs on LG9 all dropped below two, while the P-value of SNPs on the other LGs generally did not change. Similar results were obtained for other associated SNPs. Due to potential errors in genome sequence assembly, a false positive QTL could be mapped on a wrong position. The significant SNPs are independent to those on different LGs, which proved that no associated QTL was identified on a wrong LG caused by incorrect scaffolding in the reference genome sequence (Liu *et al.* 2016).

## Discussion

Head shape is important not only for understanding evolutionary adaptation, but also for aquaculture reasons of catfish. Smaller heads are beneficial for aquaculture production and profit margins. In this work, we used the high density 250K catfish SNP array and the backcross progenies for mapping QTL controlling head size.

**Figure 3:**
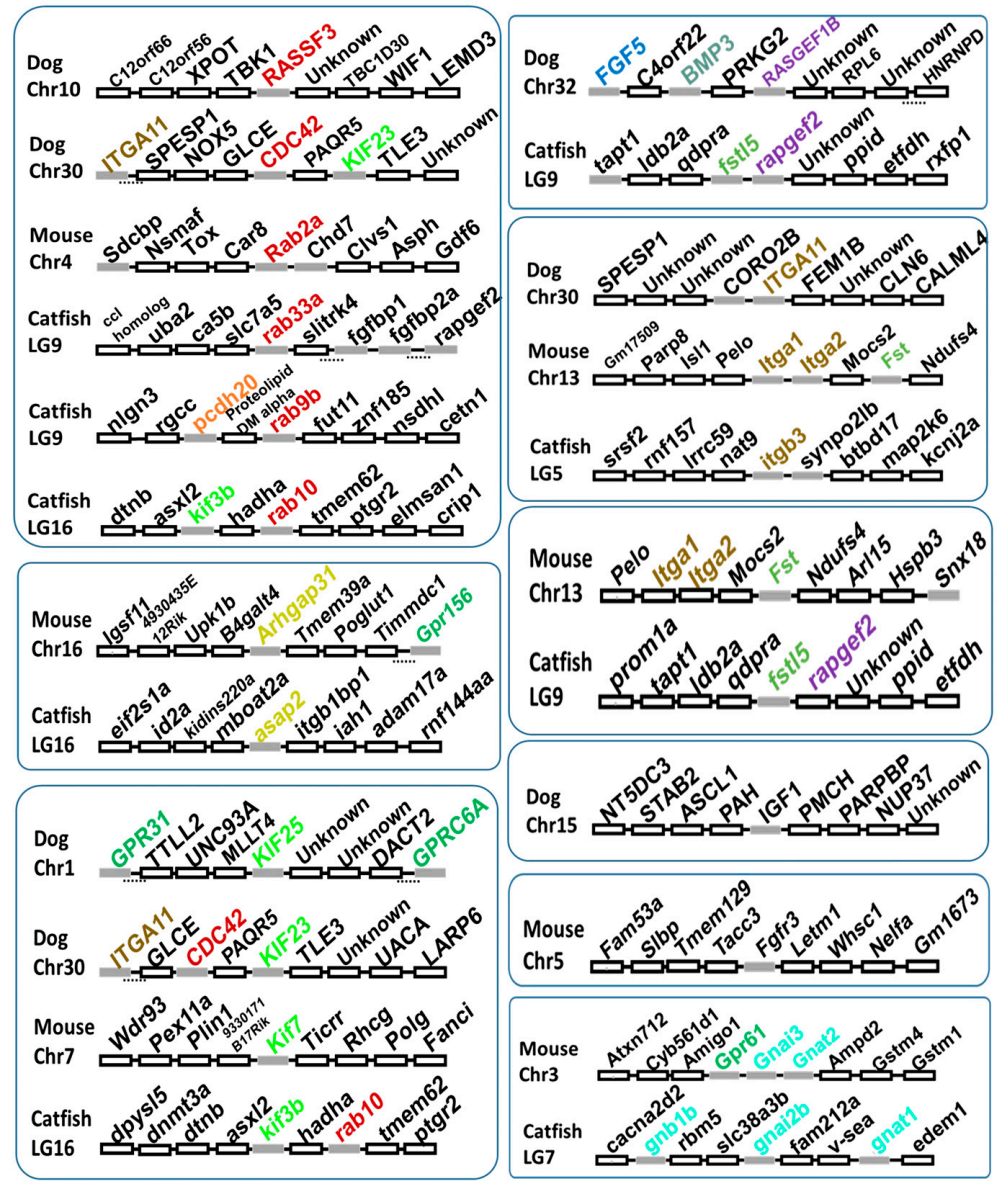
Regional scan of QTL associated with head shape identified in mouse and dog (Schoenebeck *et al.* 2012; Maga *et al.* 2015). The homologs of mouse and dog candidate genes within the associated QTL in catfish were also shown. Homologs were marked in the same color. Solid gray boxes indicate candidate genes. Dash lines under the boxes indicate several genes located in the interval are not shown. Chr, chromosome; LG, linkage group; QTL, quantitative trait loci.

Several genes were previously known to control skull shape and size in fish, frog, dog, mouse, and human, which are functionally related to the candidate genes reported here. For instance, *bmp4* was reported to play an important role in coordinating shape differences in the cichlid fish oral jaw apparatus (Albertson *et al.* 2003). It was reported that small GTPases are important to cell adhesion and head formation in early *Xenopus* development (Wunnenberg-Stapleton *et al.* 1999). In humans, mutations affecting *FGFR*, *RAB*, and *TGFBR* are associated with defects within the developing skull (Schoenebeck and Ostrander 2013). In dogs, eight QTL were reported to be associated with skull diversity, and it was demonstrated that *BMP3* contains a likely causal variant (Schoenebeck *et al.* 2012). In addition to *BMP3*, we searched the other associated regions for candidate genes surrounding the proposed significant SNPs in dogs (Schoenebeck *et al.* 2012). In doing so, many candidate genes were identified, coding for proteins including small GTPase, Ras guanine nucleotide exchange factor 1b (RASGEF1B), integrin alpha 11 (ITGA11), fibroblast growth factor 5 (FGF5), kinesin-like protein (KIF), and insulin-like growth factor 1 (IGF1) (Figure 3). It is notable that some homologs were identified associated with head size in catfish. Similarly, *Itga2*, *Arhgap31*, *Gnai3*, *Fgfr3*, *Chd7*, and *Kif7* were identified within the QTL linked with mouse skull shape (Maga *et al.* 2015). Close to *Gnai3*, *Itga2*, and *Chd7*, we also found *Gnat2*, *Itga1*, *Fst*, and *Rab2a* according to the mouse genome sequence (Figure 3). By examining the genomic regions associated with head shape in catfish, dog, and mouse (Figure 3), it is clear that all the identified genes were not orthologous. Therefore, it appears that it is not orthologous genes that explain the variance of head shape in different species. The first reason may be that different landmarks were utilized to describe the head shape in three studies. Second, maybe not all the orthologous genes contain variants that could affect the phenotype in the sampled population within three species or could be mapped, even if they are involved in head shape in the three species.

It is also notable that some paralogs, including *Ras-related protein*, *small GTPase-activating protein*, and *sphingomyelin phosphodiesterase*, were enriched in the regions associated with head size on different LGs (Table 2 and Table 3). For example, 52 Rab GTPase genes were identified out of the whole channel catfish genome (Wang *et al.* 2014), which was predicted to contain 26,661 protein-coding genes (Liu *et al.* 2016). In the nine associated QTL containing about 420 genes, three Rab genes were identified, which is more than would be expected by chance (*P* < 0.05).

In addition to the enriched Rab genes, most candidate genes are functionally related to small GTPase (Figure S6, Table 2, and Table 3). Small GTPases are reported to be involved in bone morphogenesis with a variety of functions, including the dynamics of the actin cytoskeleton, cell adhesion, and membrane trafficking (Wunnenberg-Stapleton *et al.* 1999; Itzstein *et al.* 2011). They are involved in the functions of some cell adhesion molecules, including cadherins, protocadherins, and integrins (Watanabe *et al.* 2009). Within the region associated with head length, for example, eight candidate genes on LG9 were found with functions related to head formation (Figure S3 and Table 2). One small GTPase gene and five genes with known functions highly related to small GTPase were identified in the region on LG9, including *ras-related protein rab-33a* (*rab33a*), *rap guanine nucleotide exchange factor 2* (*rapgef2*) (Quilliam *et al.* 2002), *fibroblast growth factor-binding protein-1* (*fgfbp1*) and -*2a* (*fgfbp2a*) (Szebenyi and Fallon 1998), *sphingomyelin phosphodiesterase 3* (*smpd3*) (Aubin *et al.* 2005; Tomiuk *et al.* 1998), and *septin-7* (*sept7*) (Cao *et al.* 2007; Longtine and Bi 2003) (Figure S3). Apart from these genes, the other candidate genes within the associated region are involved in the BMP pathway, which is related to small GTPase. Follistatin-related protein (FSTL) could bind actin and BMP, which is important in cartilage and bone development (Sidis *et al.* 2002). Transmembrane anterior posterior transformation 1 (Tapt1), regulated by BMP (McPherron *et al.* 1999), is speculated to be related to axial skeletal patterning during development (Howell *et al.* 2007). Within the regions associated with head width on LG9, *Rab-9b*, *sorting nexin 25* (*snx25*), *protocadherin 20* (*pcdh20*), and *actin-binding LIM protein 3* (*ablm3*) were identified. Sorting nexins are reported to interact with the BMP pathway (Parks *et al.* 2001). Protocadherins, a subgroup of the cadherin superfamily, could regulate gastrulation via small GTPase (Unterseher *et al.* 2004). Moreover, on the other associated regions, most of candidate genes are known to be related to small GTPases, implying the importance of small GTPases and the functional relationship of candidate genes (Table 2 and Table 3).

We previously proposed the “functional hubs” within QTL of columnaris resistance (Geng *et al.* 2015), where related genes in the same or similar pathways are physically together. Here, once again, strong clusters of genes involved in the same pathway were also observed for head shape QTL (Figure 3, Table 2, and Table 3) (Geng *et al.* 2015; Michalak 2008). Although it is possible that just one causal gene is involved in each of the associated regions, it is also possible, and even likely, that the candidate genes in the functional hubs work together to regulate the involved traits.

Sample populations based on large families are suitable for GWAS of most aquaculture species, because the large number of offspring per spawning in most aquaculture species could ensure enough samples at a low cost. In our study, samples based on nine families were used. Compared with unrelated samples, GWAS based on large families has some advantages in identifying QTL (Ott *et al.* 2011). First, the lack of recombination between causative variations and associated markers increases the power for detection (Mackay and Powell 2007). However, the tradeoff is that mapping resolution is reduced, which results in the long-extending regions of QTL. To narrow down the regions, local SNP markers could be selected around the identified QTL. Instead of genotyping SNPs on the whole genome, genotyping local SNPs costs less, which allows larger number of samples to be included to detect rare recombination. Thus, fine mapping and cost-effective QTL analysis could be achieved by using a two-step strategy. In order to improve brood stocks in catfish production by MAS, further analysis on local SNPs is required to provide more accurate QTL information based on our preliminary data. Second, the clear pedigree information of family-based population design makes it much easier to control the confounding factor caused by population stratification. Nevertheless, the pitfalls of family-based population should not be ignored. The family or population specification of QTL is one of the major reasons for the variance of phenotype. The limited number of founders in the family-based samples may reduce the power to detect QTL. Including more families in the samples could allow validation, increase mapping resolution, improve statistical power, and avoid false positives.

The high fecundity makes family-based samples feasible and efficient for GWAS in most aquaculture species. However, for samples consisting of large families in aquaculture, the performances of commonly used test methods have not been compared. In our study, two methods, EMMAX and QFAM, were evaluated for family-based samples. EMMAX and QFAM are both effective in correcting population stratification. Population stratification is the major confounding factor causing false positive results. If the population stratification is not corrected, false positive results that are associated with population structure rather than the trait of interest could be detected. QFAM is just applicable for family-based population to control population stratification. QFAM partitions the genotypes into between- and within- family components (Fulker *et al.* 1999; Abecasis *et al.* 2000). The within-family components could control stratification, and the true association results could be identified without the effect from stratification. Unlike QFAM, EMMAX calculates a pairwise relatedness matrix according to high-density markers to represent the sample structure at first. Then, EMMAX can estimate the contribution of the sample structure to the phenotype and detect associations without confounding effects generated from sample structure (Kang *et al.* 2010). EMMAX has been proven to be widely applicable for correcting family structure, as well as population structure and cryptic relatedness (Price *et al.* 2010). However, it has been shown that inclusion of the candidate markers to calculate the pairwise relatedness matrix could lead to loss in power because of double-fitting of the candidate markers in the model (Yang *et al.* 2014). In our study, EMMAX has less power compared with QFAM. Due to the relatively low power of EMMAX, we concluded that, in terms of statistical power, QFAM performs better than EMMAX with large families in our study.

Our long-term goal is to enhance catfish stocks with a favorable phenotype for head shape, incorporate this trait along with other traits such as disease resistance, and finally support a sustainable and profitable aquaculture industry. Parental fish with homozygous favorable alleles can be screened, making them immediately applicable to the catfish industry. To reach this long-term goal, the genetic basis underlying such traits must be understood, especially the accurate location of QTL controlling the traits of interest. Detailed QTL information will then be used to improve brood stocks by MAS, or introgression of valuable disease resistance alleles from both channel catfish and blue catfish. In our study, GWAS could locate part of associated QTL into several Mb, so fine mapping of QTL is still needed.

### Conclusion

This study investigated the genetic basis of head size of catfish backcross fingerlings. Several QTL were identified to be associated with head length and head width. Many genes related to bone development were identified within these associated regions in catfish. Homologs of several candidate genes are involved in skull morphology in various other species ranging from amphibian to mammalian species.

## Supplementary Material

Supplemental Material

## References

[bib1] Abecasis G. Cardon L. Cookson W., 2000 A general test of association for quantitative traits in nuclear families. Am. J. Hum. Genet. 66(1): 279–292. 10631157 10.1086/302698 PMC1288332

[bib2] Albertson R. C. Streelman J. T. Kocher T. D., 2003 Directional selection has shaped the oral jaws of Lake Malawi cichlid fishes. Proc. Natl. Acad. Sci. USA 100(9): 5252–5257. 12704237 10.1073/pnas.0930235100 PMC154331

[bib3] Altschul S. F. Gish W. Miller W. Myers E. W. Lipman D. J., 1990 Basic local alignment search tool. J. Mol. Biol. 215(3): 403–410. 2231712 10.1016/S0022-2836(05)80360-2

[bib4] Argue B. J. Liu Z. Dunham R. A., 2003 Dress-out and fillet yields of channel catfish, *Ictalurus punctatus*, blue catfish, *Ictalurus furcatus*, and their F 1, F 2 and backcross hybrids. Aquaculture 228(1): 81–90.

[bib5] Asanuma K. Yanagida-Asanuma E. Faul C. Tomino Y. Kim K., 2006 Synaptopodin orchestrates actin organization and cell motility via regulation of RhoA signalling. Nat. Cell Biol. 8(5): 485–491. 16622418 10.1038/ncb1400

[bib6] Aubin I. Adams C. P. Opsahl S. Septier D. Bishop C. E., 2005 A deletion in the gene encoding sphingomyelin phosphodiesterase 3 (Smpd3) results in osteogenesis and dentinogenesis imperfecta in the mouse. Nat. Genet. 37(8): 803–805. 16025116 10.1038/ng1603

[bib7] Ayllon F. Kjærner-Semb E. Furmanek T. Wennevik V. Solberg M. F., 2015 The vgll3 locus controls age at maturity in wild and domesticated Atlantic salmon (*Salmo salar L*.) males. PLoS Genet. 11(11): e1005628. 26551894 10.1371/journal.pgen.1005628 PMC4638356

[bib8] Cao L. Ding X. Yu W. Yang X. Shen S., 2007 Phylogenetic and evolutionary analysis of the septin protein family in metazoan. FEBS Lett. 581(28): 5526–5532. 17967425 10.1016/j.febslet.2007.10.032

[bib9] Correa K. Lhorente J. P. López M. E. Bassini L. Naswa S., 2015 Genome-wide association analysis reveals loci associated with resistance against *Piscirickettsia salmonis* in two Atlantic salmon (*Salmo salar L*.) chromosomes. BMC Genomics 16(1): 1. 26499328 10.1186/s12864-015-2038-7 PMC4619534

[bib10] Correa K. Lhorente J. P. Bassini L. López M. E. Di Genova A., 2016 Genome wide association study for resistance to *Caligus rogercresseyi* in Atlantic salmon (*Salmo salar* L.) using a 50K SNP genotyping array. Aquaculture. DOI: 10.1016/j.aquaculture.2016.04.008.

[bib11] Deng C. Wynshaw-Boris A. Zhou F. Kuo A. Leder P., 1996 Fibroblast growth factor receptor 3 is a negative regulator of bone growth. Cell 84(6): 911–921. 8601314 10.1016/s0092-8674(00)81069-7

[bib12] Dos Remedios C. Chhabra D. Kekic M. Dedova I. Tsubakihara M., 2003 Actin binding proteins: regulation of cytoskeletal microfilaments. Physiol. Rev. 83(2): 433–473. 12663865 10.1152/physrev.00026.2002

[bib13] Dunham, R. A., and R. O. Smitherman, 1984 Ancestry and breeding of catfish in the United States. Alabama Agricultural Experiment Station, Auburn, AL.

[bib14] Dunham R. A. Umali G. M. Beam R. Kristanto A. H. Trask M., 2008 Comparison of production traits of NWAC103 channel catfish, NWAC103 channel catfish× blue catfish hybrids, Kansas Select 21 channel catfish, and blue catfish grown at commercial densities and exposed to natural bacterial epizootics. N. Am. J. Aquaculture 70(1): 98–106.

[bib15] Froese R., 2006 Cube law, condition factor and weight–length relationships: history, meta‐analysis and recommendations. J. Appl. Ichthyology 22(4): 241–253.

[bib16] Fulker D. Cherny S. Sham P. Hewitt J., 1999 Combined linkage and association sib-pair analysis for quantitative traits. Am. J. Hum. Genet. 64(1): 259–267. 9915965 10.1086/302193 PMC1377724

[bib17] Geng X. Sha J. Liu S. Bao L. Zhang J., 2015 A genome-wide association study in catfish reveals the presence of functional hubs of related genes within QTLs for columnaris disease resistance. BMC Genomics 16(1): 196. 25888203 10.1186/s12864-015-1409-4 PMC4372039

[bib18] Gong H. Shen B. Flevaris P. Chow C. Lam S. C.-T., 2010 G protein subunit Gα13 binds to integrin αIIbβ3 and mediates integrin “outside-in” signaling. Science 327(5963): 340–343. 20075254 10.1126/science.1174779 PMC2842917

[bib19] Gutierrez A. P. Yáñez J. M. Fukui S. Swift B. Davidson W. S., 2015 Genome-wide association study (GWAS) for growth rate and age at sexual maturation in Atlantic salmon (*Salmo salar*). PLoS One 10(3): e0119730. 25757012 10.1371/journal.pone.0119730 PMC4355585

[bib20] Homme M. Schmitt C. Himmele R. Hoffmann G. Mehls O., 2003 Vitamin D and dexamethasone inversely regulate parathyroid hormone-induced regulator of G protein signaling-2 expression in osteoblast-like cells. Endocrinology 144(6): 2496–2504. 12746312 10.1210/en.2002-0160

[bib21] Howell G. R. Shindo M. Murray S. Gridley T. Wilson L. A., 2007 Mutation of a ubiquitously expressed mouse transmembrane protein (Tapt1) causes specific skeletal homeotic transformations. Genetics 175(2): 699–707. 17151244 10.1534/genetics.106.065177 PMC1800629

[bib22] Itzstein C. Coxon F. P. Rogers M. J., 2011 The regulation of osteoclast function and bone resorption by small GTPases. Small GTPases 2(3): 117–130. 21776413 10.4161/sgtp.2.3.16453 PMC3136942

[bib23] Jin Y. Zhou T. Geng X. Liu S. Chen A., 2016 A genome‐wide association study of heat stress‐associated SNPs in catfish. Anim. Genet. 10.1111/age.12482. 27476875

[bib24] Kang H. M. Sul J. H. Service S. K. Zaitlen N. A. Kong S.-y., 2010 Variance component model to account for sample structure in genome-wide association studies. Nat. Genet. 42(4): 348–354. 20208533 10.1038/ng.548 PMC3092069

[bib25] Kucuktas H. Wang S. Li P. He C. Xu P., 2009 Construction of genetic linkage maps and comparative genome analysis of catfish using gene-associated markers. Genetics 181(4): 1649–1660. 19171943 10.1534/genetics.108.098855 PMC2666527

[bib26] Laghari M. Lashari P. Zhang X. Xu P. Xin B., 2014 Mapping quantitative trait loci (QTL) for body weight, length and condition factor traits in backcross (BC1) family of Common carp (*Cyprinus carpio L*.). Mol. Biol. Rep. 41(2): 721–731. 24368591 10.1007/s11033-013-2911-x

[bib27] Liu S. Sun L. Li Y. Sun F. Jiang Y., 2014 Development of the catfish 250K SNP array for genome-wide association studies. BMC Res. Notes 7(1): 135. 24618043 10.1186/1756-0500-7-135 PMC3995806

[bib28] Liu Z. Karsi A. Li P. Cao D. Dunham R., 2003 An AFLP-based genetic linkage map of channel catfish (*Ictalurus punctatus*) constructed by using an interspecific hybrid resource family. Genetics 165(2): 687–694. 14573480 10.1093/genetics/165.2.687 PMC1462775

[bib29] Liu Z. Liu S. Yao J. Bao L. Zhang J., 2016 The channel catfish genome sequence provides insights into the evolution of scale formation in teleosts. Nat. Commun. 7: 11757. 27249958 10.1038/ncomms11757 PMC4895719

[bib30] Longtine M. S. Bi E., 2003 Regulation of septin organization and function in yeast. Trends Cell Biol. 13(8): 403–409. 12888292 10.1016/s0962-8924(03)00151-x

[bib31] Mackay I. Powell W., 2007 Methods for linkage disequilibrium mapping in crops. Trends Plant Sci. 12(2): 57–63. 17224302 10.1016/j.tplants.2006.12.001

[bib32] Maga A. M. Navarro N. Cunningham M. L. Cox T. C., 2015 Quantitative trait loci affecting the 3D skull shape and size in mouse and prioritization of candidate genes in-silico. Front. Physiol. 6: 92. 25859222 10.3389/fphys.2015.00092 PMC4374467

[bib33] Marie P. J., 2002 Role of N‐cadherin in bone formation. J. Cell. Physiol. 190(3): 297–305. 11857445 10.1002/jcp.10073

[bib34] Massault C. Hellemans B. Louro B. Batargias C. Van Houdt J., 2010 QTL for body weight, morphometric traits and stress response in European sea bass *Dicentrarchus labrax*. Anim. Genet. 41(4): 337–345. 20028379 10.1111/j.1365-2052.2009.02010.x

[bib35] McDonald A. Schuijers J. Gundlach A. Grills B., 2007 Galanin treatment offsets the inhibition of bone formation and downregulates the increase in mouse calvarial expression of TNFα and GalR2 mRNA induced by chronic daily injections of an injurious vehicle. Bone 40(4): 895–903. 17157570 10.1016/j.bone.2006.10.018

[bib36] McPherron A. C. Lawler A. M. Lee S.-J., 1999 Regulation of anterior/posterior patterning of the axial skeleton by growth/differentiation factor 11. Nat. Genet. 22(3): 260–264. 10391213 10.1038/10320

[bib37] Michalak P., 2008 Coexpression, coregulation, and cofunctionality of neighboring genes in eukaryotic genomes. Genomics 91(3): 243–248. 18082363 10.1016/j.ygeno.2007.11.002

[bib38] Myers K. R. Casanova J. E., 2008 Regulation of actin cytoskeleton dynamics by Arf-family GTPases. Trends Cell Biol. 18(4): 184–192. 18328709 10.1016/j.tcb.2008.02.002 PMC2885709

[bib39] Ninwichian P. Peatman E. Liu H. Kucuktas H. Somridhivej B., 2012 Second-generation genetic linkage map of catfish and its integration with the BAC-based physical map. G3 (Bethesda) 2(10):1233–1241. 23050234 10.1534/g3.112.003962 PMC3464116

[bib40] Ott J. Kamatani Y. Lathrop M., 2011 Family-based designs for genome-wide association studies. Nat. Rev. Genet. 12(7): 465–474. 21629274 10.1038/nrg2989

[bib41] Parks W. T. Frank D. B. Huff C. Haft C. R. Martin J., 2001 Sorting nexin 6, a novel SNX, interacts with the transforming growth factor-β family of receptor serine-threonine kinases. J. Biol. Chem. 276(22): 19332–19339. 11279102 10.1074/jbc.M100606200

[bib42] Price A. L. Zaitlen N. A. Reich D. Patterson N., 2010 New approaches to population stratification in genome-wide association studies. Nat. Rev. Genet. 11(7): 459–463. 20548291 10.1038/nrg2813 PMC2975875

[bib43] Pruitt K. D. Tatusova T. Maglott D. R., 2007 NCBI reference sequences (RefSeq): a curated non-redundant sequence database of genomes, transcripts and proteins. Nucleic Acids Res. 35(Suppl. 1): D61–D65. 17130148 10.1093/nar/gkl842 PMC1716718

[bib44] Purcell S. Neale B. Todd-Brown K. Thomas L. Ferreira M. A., 2007 PLINK: a tool set for whole-genome association and population-based linkage analyses. Am. J. Hum. Genet. 81(3): 559–575. 17701901 10.1086/519795 PMC1950838

[bib45] Quarles L. Siddhanti S., 1996 Guanine nucleotide binding‐protein coupled signaling pathway regulation of osteoblast‐mediated bone formation. J. Bone Miner. Res. 11(10): 1375–1383. 8889835 10.1002/jbmr.5650111002

[bib46] Quilliam L. A. Rebhun J. F. Castro A. F., 2002 A growing family of guanine nucleotide exchange factors is responsible for activation of Ras-family GTPases. Prog. Nucleic Acid Res. Mol. Biol. 71: 391–444. 12102558 10.1016/s0079-6603(02)71047-7

[bib47] Ross F. Chappel J. Alvarez J. Sander D. Butler W., 1993 Interactions between the bone matrix proteins osteopontin and bone sialoprotein and the osteoclast integrin alpha v beta 3 potentiate bone resorption. J. Biol. Chem. 268(13): 9901–9907. 8486670

[bib48] Rutten M. J. Bovenhuis H. Komen H., 2005 Genetic parameters for fillet traits and body measurements in Nile tilapia (*Oreochromis niloticus L*.). Aquaculture 246(1): 125–132.

[bib49] Schoenebeck J. J. Ostrander E. A., 2013 The genetics of canine skull shape variation. Genetics 193(2): 317–325. 23396475 10.1534/genetics.112.145284 PMC3567726

[bib50] Schoenebeck J. J. Hutchinson S. A. Byers A. Beale H. C. Carrington B., 2012 Variation of BMP3 contributes to dog breed skull diversity. PLoS Genet. 8(8): e1002849. 22876193 10.1371/journal.pgen.1002849 PMC3410846

[bib51] Shears S. B. Gokhale N. A. Wang H. Zaremba A., 2011 Diphosphoinositol polyphosphates: what are the mechanisms? Adv. Enzyme Regul. 51(1): 13. 21035493 10.1016/j.advenzreg.2010.09.008 PMC3507380

[bib52] Sidis Y. Tortoriello D. V. Holmes W. E. Pan Y. Keutmann H. T., 2002 Follistatin-related protein and follistatin differentially neutralize endogenous *vs.* exogenous activin. Endocrinology 143(5): 1613–1624. 11956142 10.1210/endo.143.5.8805

[bib53] Sodeland M. Gaarder M. Moen T. Thomassen M. Kjøglum S., 2013 Genome-wide association testing reveals quantitative trait loci for fillet texture and fat content in Atlantic salmon. Aquaculture 408: 169–174.

[bib54] Solovyev V. Kosarev P. Seledsov I. Vorobyev D., 2006 Automatic annotation of eukaryotic genes, pseudogenes and promoters. Genome Biol. 7(Suppl. 1): S10. 16925832 10.1186/gb-2006-7-s1-s10 PMC1810547

[bib55] St-Arnaud R. Naja R. P., 2011 Vitamin D metabolism, cartilage and bone fracture repair. Mol. Cell. Endocrinol. 347(1): 48–54. 21664253 10.1016/j.mce.2011.05.018

[bib56] Sun L. Liu S. Wang R. Jiang Y. Zhang Y., 2014 Identification and analysis of genome-wide SNPs provide insight into signatures of selection and domestication in channel catfish (*Ictalurus punctatus*). PLoS One 9(10): e109666. 25313648 10.1371/journal.pone.0109666 PMC4196944

[bib57] Sutter N. B. Bustamante C. D. Chase K. Gray M. M. Zhao K., 2007 A single IGF1 allele is a major determinant of small size in dogs. Science 316(5821): 112–115. 17412960 10.1126/science.1137045 PMC2789551

[bib58] Szebenyi G. Fallon J. F., 1998 Fibroblast growth factors as multifunctional signaling factors. Int. Rev. Cytol. 185: 45–106. 10.1016/s0074-7696(08)60149-7 9750265

[bib59] Thirunavukkarasu K. Halladay D. L. Miles R. R. Geringer C. D. Onyia J. E., 2002 Analysis of regulator of G‐protein signaling‐2 (RGS‐2) expression and function in osteoblastic cells. J. Cell. Biochem. 85(4): 837–850. 11968023 10.1002/jcb.10176

[bib60] Tomiuk S. Hofmann K. Nix M. Zumbansen M. Stoffel W., 1998 Cloned mammalian neutral sphingomyelinase: Functions in sphingolipid signaling? Proc. Natl. Acad. Sci. USA 95(7): 3638–3643. 9520418 10.1073/pnas.95.7.3638 PMC19888

[bib61] Tsai H.-Y. Hamilton A. Tinch A. E. Guy D. R. Gharbi K., 2015 Genome wide association and genomic prediction for growth traits in juvenile farmed Atlantic salmon using a high density SNP array. BMC Genomics 16(1): 969. 26582102 10.1186/s12864-015-2117-9 PMC4652364

[bib62] Tsai H.-Y. Hamilton A. Tinch A. E. Guy D. R. Bron J. E., 2016 Genomic prediction of host resistance to sea lice in farmed Atlantic salmon populations. Genet. Sel. Evol. 48(1): 1. 27357694 10.1186/s12711-016-0226-9 PMC4926294

[bib63] Unterseher F. Hefele J. A. Giehl K. De Robertis E. M. Wedlich D., 2004 Paraxial protocadherin coordinates cell polarity during convergent extension via Rho A and JNK. EMBO J. 23(16): 3259–3269. 15297873 10.1038/sj.emboj.7600332 PMC514506

[bib64] Wang R. Zhang Y. Liu S. Li C. Sun L., 2014 Analysis of 52 Rab GTPases from channel catfish and their involvement in immune responses after bacterial infections. Dev. Comp. Immunol. 45(1): 21–34. 24513270 10.1016/j.dci.2014.01.026

[bib65] Watanabe T. Sato K. Kaibuchi K., 2009 Cadherin-mediated intercellular adhesion and signaling cascades involving small GTPases. Cold Spring Harb. Perspect. Biol. 1(3): a003020. 20066109 10.1101/cshperspect.a003020 PMC2773633

[bib66] Wunnenberg-Stapleton K. Blitz I. L. Hashimoto C. Cho K., 1999 Involvement of the small GTPases XRhoA and XRnd1 in cell adhesion and head formation in early Xenopus development. Development 126(23): 5339–5351. 10556059 10.1242/dev.126.23.5339

[bib67] Yang J. Zaitlen N. A. Goddard M. E. Visscher P. M. Price A. L., 2014 Advantages and pitfalls in the application of mixed-model association methods. Nat. Genet. 46(2): 100–106. 24473328 10.1038/ng.2876 PMC3989144

[bib68] Zhao H. Ito Y. Chappel J. Andrews N. W. Teitelbaum S. L., 2008 Synaptotagmin VII regulates bone remodeling by modulating osteoclast and osteoblast secretion. Dev. Cell 14(6): 914–925. 18539119 10.1016/j.devcel.2008.03.022 PMC2480494

