# Supplementary material for: A Genome-Wide Association Study Identifies Multiple Regions Associated with Head Size in Catfish
Source: G3 (Bethesda). 2016 Aug 24;6(10):3389–98. doi: 10.1534/g3.116.032201 (PMC5068958; doi:10.1534/g3.116.032201)
Supplement: Supplemental Material [file supp_6_10_3389__index.html]

A Genome-Wide Association Study Identifies Multiple Regions Associated with Head Size in Catfish — Supplemental Material 

# A Genome-Wide Association Study Identifies Multiple Regions Associated with Head Size in Catfish

## Supplemental Material for Geng *et al.*, 2016

**Files in this Data Supplement:**

- Figure S1 - Sample structure identified by PCA with the first three principal components using sample genotypes. (.pdf, 184 KB)
- Figure S2 - Morphometric measurement of catfish skull. (.pdf, 89 KB)
- Figure S3 - Regional genome scan for the QTL significantly associated with head length on LG9. (.pdf, 113 KB)
- Figure S4 - Regional genome scan for the QTL significantly associated with head width on LG9. (.pdf 89 KB)
- Figure S5 - Manhattan plots for head depth. (.pdf, 641 KB)
- Figure S6 - Signal transduction involving small GTPases and the other candidate genes. (.pdf, 89 KB)
- Table S1 - The pedigree information of catfish samples used in this study. (.xlsx, 9 KB)
