# Supplementary material for: A Genome-Wide Association Study Identifies Multiple Regions Associated with Head Size in Catfish
Source: G3 (Bethesda). 2016 Aug 24;6(10):3389–98. doi: 10.1534/g3.116.032201 (PMC5068958; doi:10.1534/g3.116.032201)
Supplement: Supplemental Material [file supp_g3.116.032201_FigureS1.pdf]

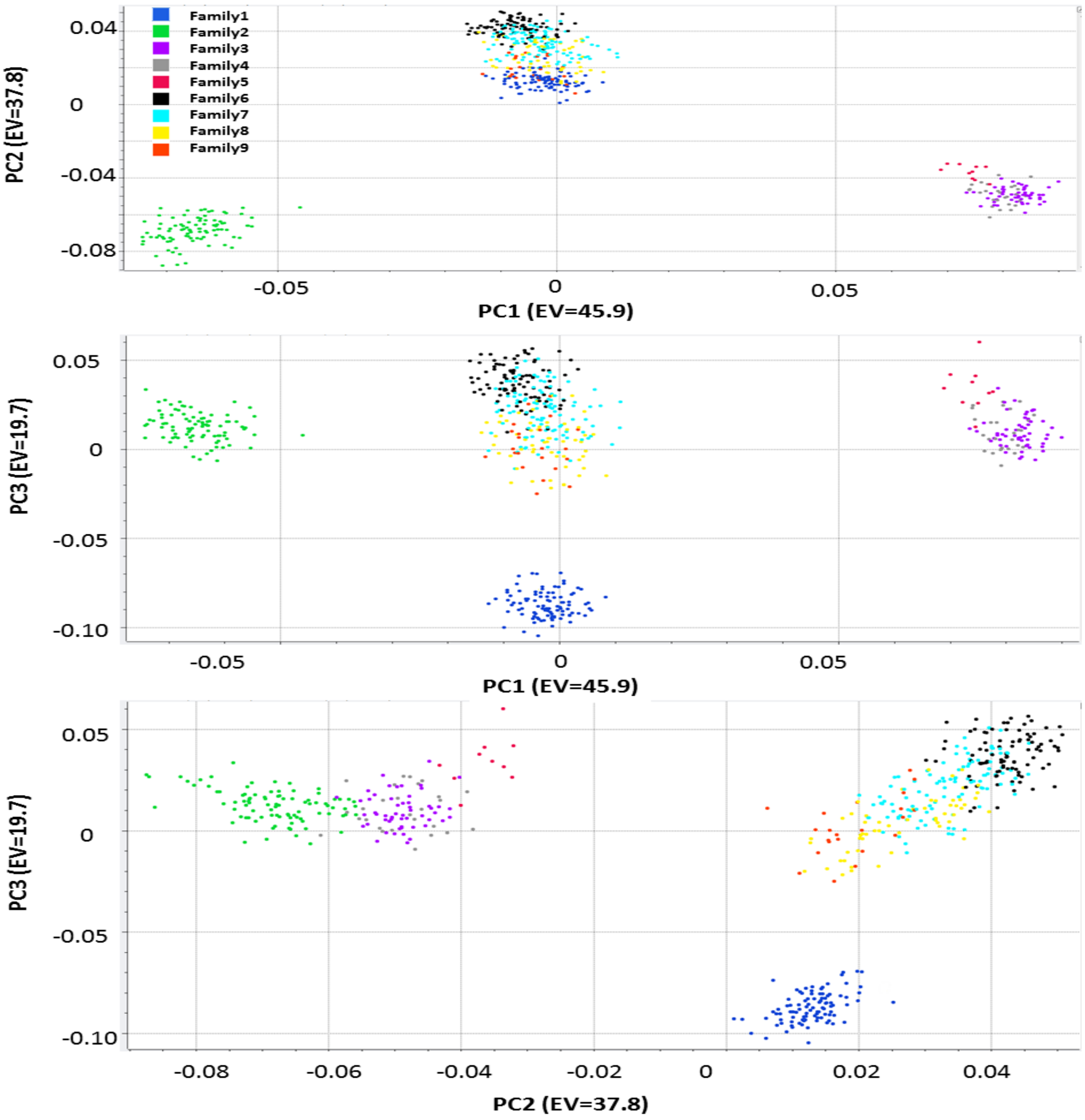

Figure S1.
Sample structure identified by PCA with the first three principal components using sample genotypes.
EV: eigenvalue.
