# Supplementary figures and images for: A Genome-Wide Association Study Identifies Multiple Regions Associated with Head Size in Catfish
Source: G3 (Bethesda). 2016 Aug 24;6(10):3389–98. doi: 10.1534/g3.116.032201 (PMC5068958; doi:10.1534/g3.116.032201)

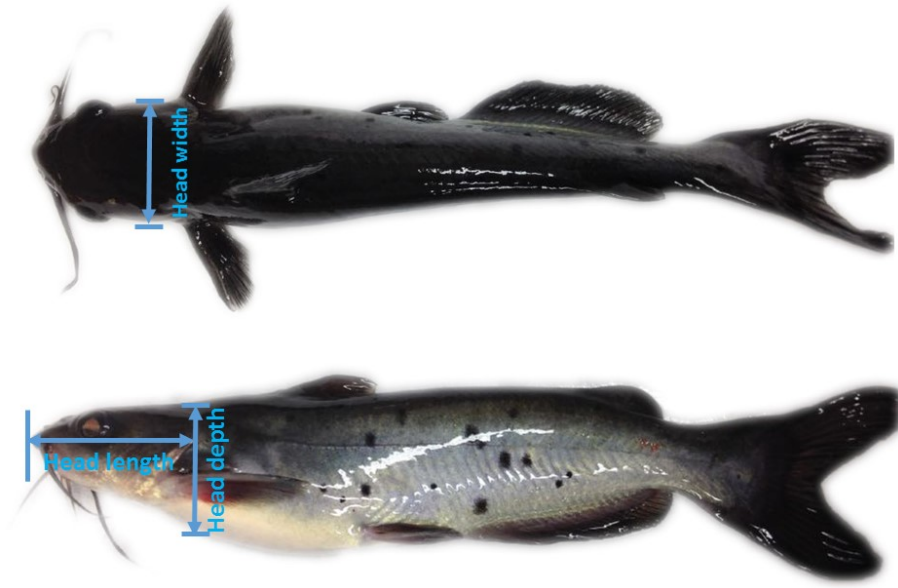

**Figure S2. Morphometric measurement of catfish skull.**

Supplement: Supplemental Material [file supp_g3.116.032201_FigureS2.pdf]

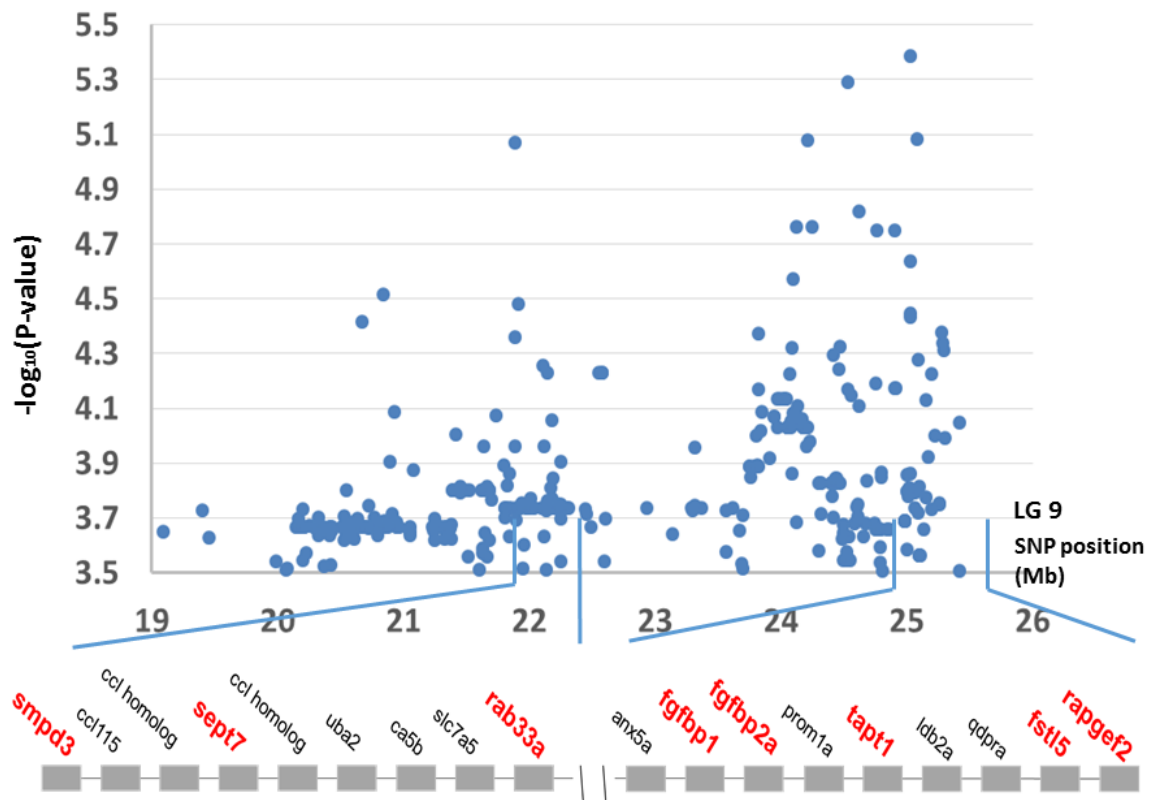

Figure S3. Regional genome scan for the QTL significantly associated with head length on LG9.

Supplement: Supplemental Material [file supp_g3.116.032201_FigureS3.pdf]

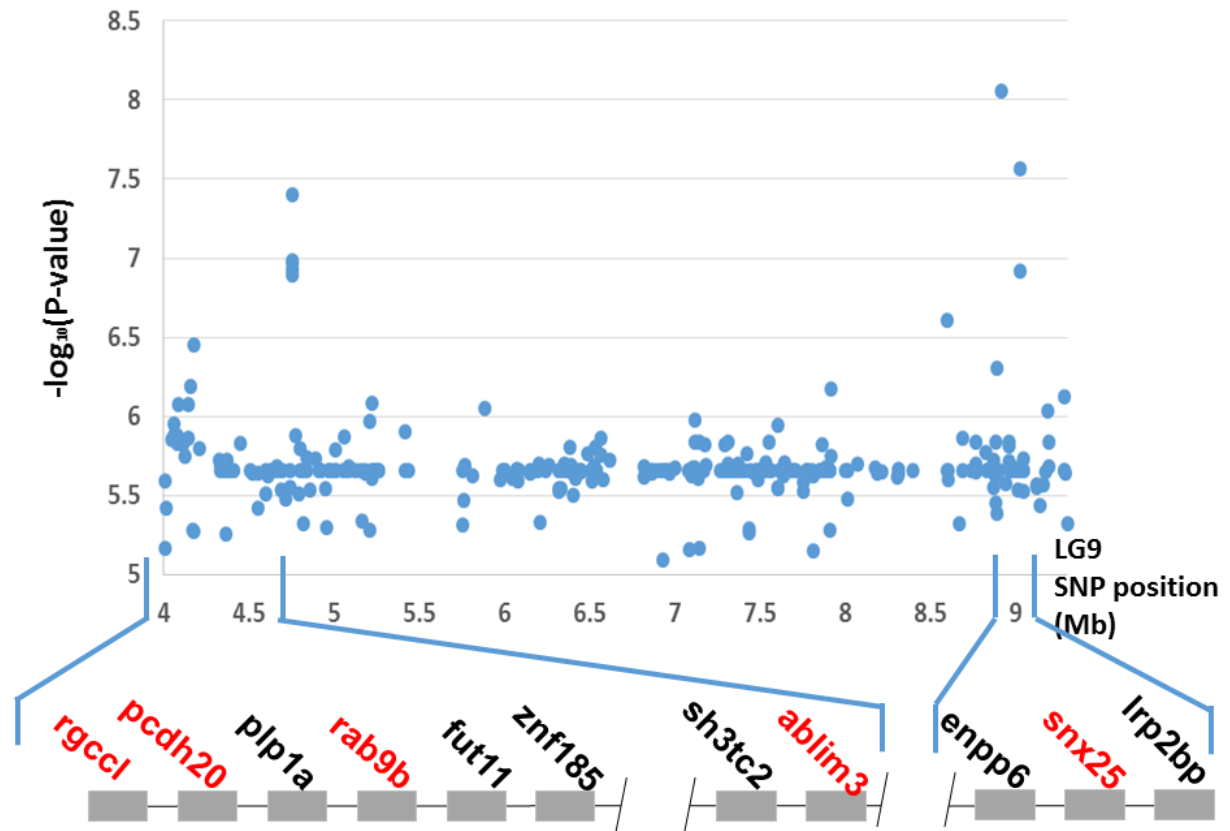

Figure S4. Regional genome scan for the QTL significantly associated with head width on LG9.

Supplement: Supplemental Material [file supp_g3.116.032201_FigureS4.pdf]

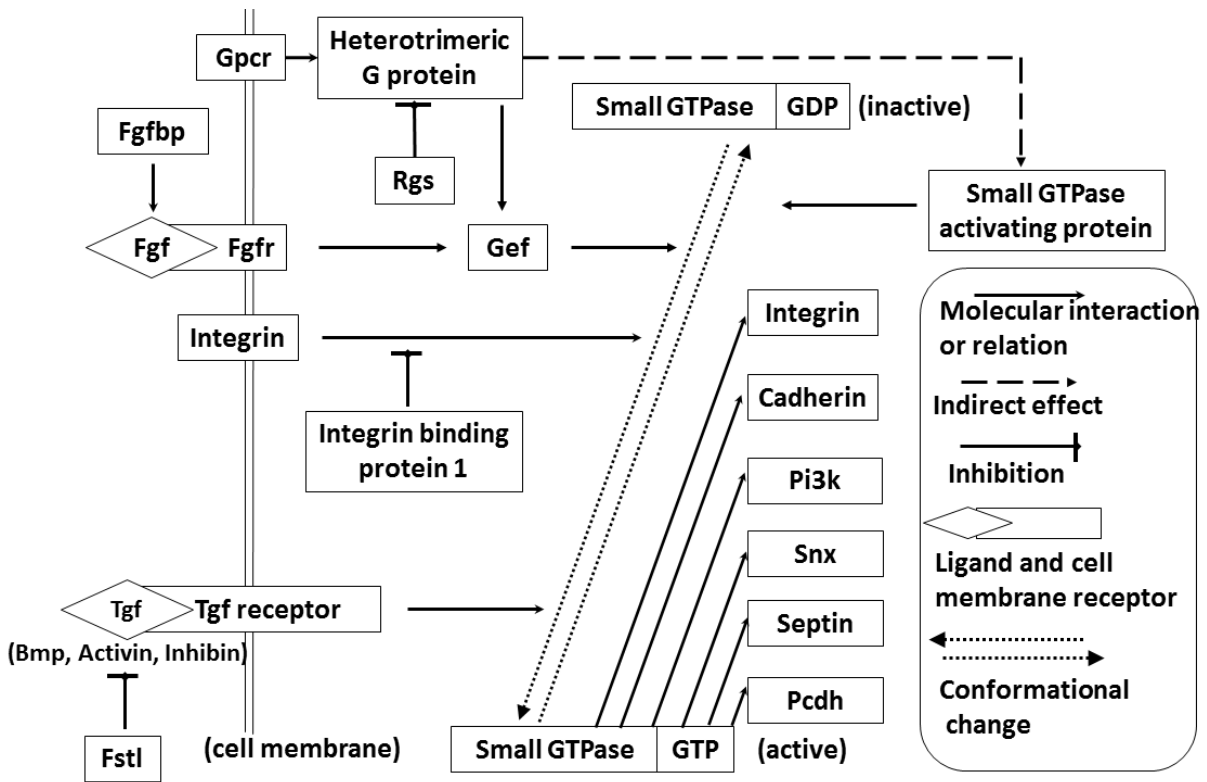

Figure S6. Signal transduction involving small GTPases and the other candidate genes.

Supplement: Supplemental Material [file supp_g3.116.032201_FigureS6.pdf]
