# Supplementary material for: A Genome-Wide Association Study Identifies Multiple Regions Associated with Head Size in Catfish
Source: G3 (Bethesda). 2016 Aug 24;6(10):3389–98. doi: 10.1534/g3.116.032201 (PMC5068958; doi:10.1534/g3.116.032201)
Supplement: Supplemental Material [file supp_g3.116.032201_FigureS5.pdf]

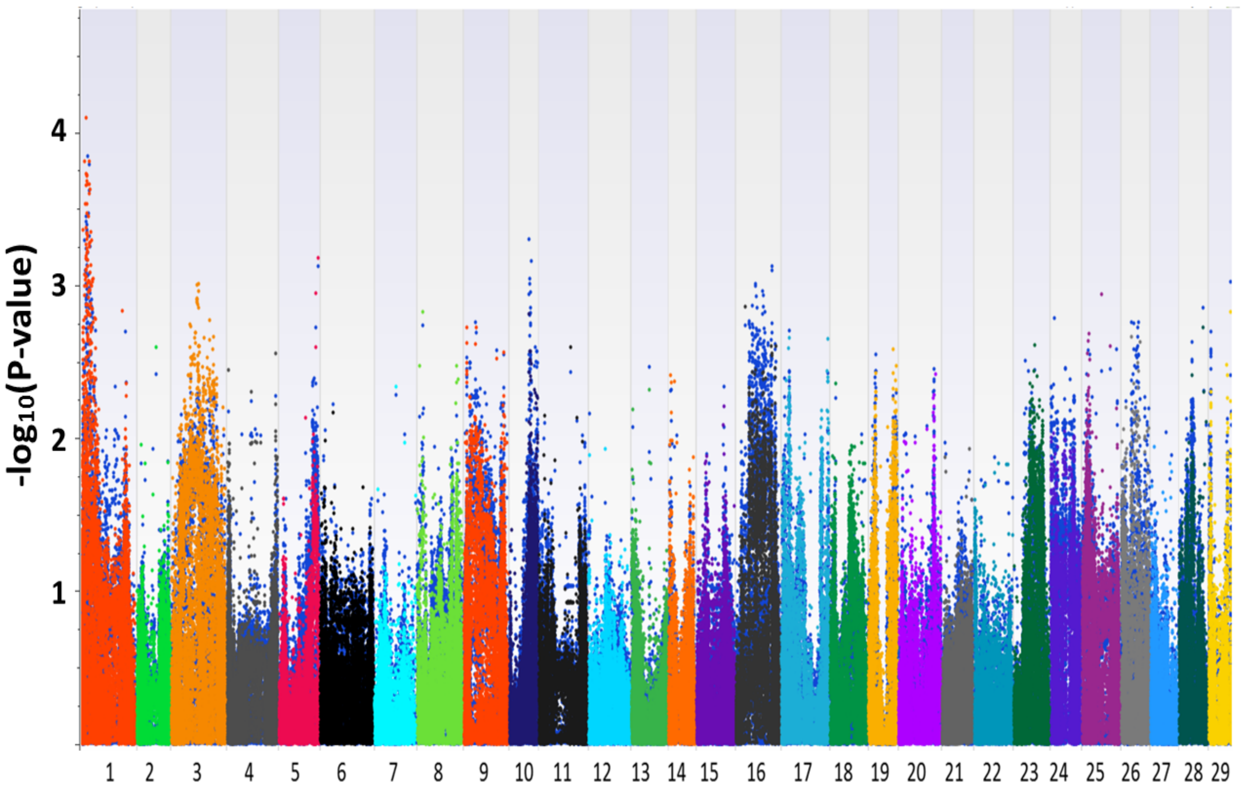

Figure S5. Manhattan plots for head depth. The plots in different colors in the front layer were generated from EMMAX and the plots in blue in the back layer were generated from QFAM.
